# The Clinical and Economic Burden of Colorectal Anastomotic Leaks: Middle-Income Country Perspective

**DOI:** 10.1155/2019/2879049

**Published:** 2019-04-01

**Authors:** Ulysses Ribeiro Jr., Daiane O. Tayar, Rodrigo A. Ribeiro, Priscila Andrade, Silvio M. Junqueira Jr.

**Affiliations:** ^1^Universidade de São Paulo Faculdade de Medicina, Brazil; ^2^Health Economics and Market Access Department, Johnson & Johnson Medical Devices Brazil, Brazil; ^3^HTAnalyze Consulting, Brazil

## Abstract

**Purpose:**

Anastomotic leaks (AL) present a significant source of clinical and economic burden on patients undergoing colorectal surgeries. This study was aimed at evaluating the clinical and economic consequences of AL and its risk factors.

**Methods:**

A retrospective cohort study was conducted between 2012 and 2013 based on the billing information of 337 patients who underwent low anterior resection (LAR). The outcomes evaluated were the development of AL, use of antibiotics, 30-day readmission and mortality, and total hospital costs, including readmissions and length of stay (LOS). The risk factors for AL, as well as the relationship between AL and clinical outcomes, were analyzed using multivariable Poisson regression. Generalized linear models (GLM) were employed to evaluate the association between AL and continuous outcomes (LOS and costs).

**Results:**

AL was detected in 6.8% of the patients. Emergency surgery (aRR 2.56; 95% CI: 1.15–5.71, *p* = 0.021), blood transfusion (aRR 4.44; 95% CI: 1.86–10.64, *p* = 0.001), and cancer diagnosis (aRR 2.51; 95% CI: 1.27–4.98, *p* = 0.008) were found to be independent predictors of AL. Patients with AL showed higher antibiotic usage (aRR 1.69; 95% CI: 1.37–2.09, *p* < 0.001), 30-day readmission (aRR 3.34; 95% CI: 1.53–7.32, *p* = 0.003) and mortality (aRR 13.49; 95% CI: 4.10–44.35, *p* < 0.001), and longer LOS (39.6 days, as opposed to 7.5 days for patients without AL, *p* < 0.001). Total hospital costs amounted to R$210,105 for patients with AL in comparison with R$34,270 for patients without AL (*p* < 0.001). In multivariable GLM, the total hospital costs for AL patients were 4.66 (95% CI: 3.38–6.23, *p* < 0.001) times higher than those for patients without AL.

**Conclusions:**

AL leads to worse clinical outcomes and increases hospital costs by 4.66 times. The risk factors for AL were found to be emergency surgery, blood transfusion, and cancer diagnosis.

## 1. Introduction

An anastomotic leak (AL) is one of the most serious surgical complications that can develop following colorectal surgery [1]. In two recent systematic reviews, the AL rate varied from 4% to 9% [2, 3]. In the review that considered only low anterior resection (LAR) studies, the pooled overall rate of AL was found to be 8.88% [3]. Several risk factors for the occurrence of AL have been identified, including male sex, diabetes mellitus, use of tobacco, blood loss, perioperative transfusion, operative time, preoperative chemoradiation, previous abdominal surgery, and emergency surgery [3–17]. On the other hand, protective stoma could reduce the occurrence of AL or reduce the severity of the AL complication [16, 17], although its routine usage shows contradictory results [18].

AL has both clinical and economic consequences. Patients with AL have longer hospital length of stay (LOS), larger probability of infection, and higher intensive care unit (ICU) admission, hospital readmission, and mortality rates [7, 19]. All these factors, among the other morbidities associated with AL, incur increased costs in this subset of patients [6, 20, 21]. With regard to the economic outcomes, the cost impact of AL varies across different economies. Recent studies have shown that the AL could increase total hospitalization costs by 0.5–1.9 times [6, 20].

The International Study Group of Rectal Cancer proposed a severity grading of AL following anterior rectal resections: grade A consists of the development of an AL treated conservatively, grade B requires invasive treatment but no surgery, and grade C requires reoperation [22].

Although there is information about the clinical and economic burden of AL in the literature, data continues to be scarce, especially in middle-income countries where financial burden poses greater challenges due to the resource constraints in such countries. The objective of this study was to evaluate the clinical and economic burden of AL in LAR in the Brazilian private healthcare system and investigate the risk factors for such outcomes.

## 2. Materials and Methods

### 2.1. Study Design and Dataset

The dataset of patients who consecutively underwent LAR between January 2012 and December 2013 was reviewed retrospectively. The administrative database of resource utilization from the Brazilian private healthcare system includes admissions, diagnoses, procedures, medications, transfusions, laboratory and imaging tests, the materials used during the patient's hospitalization, and the cost of hospitalization. Approximately 12 million patients—which account for nearly 25% of the total patients in the Brazilian private healthcare system—are included in this administrative database. The tracking of the patients in this database is done based on their health insurance plan codes and not from specific hospitals, which provides reliable follow-up data in the short term, even in cases of readmission into different hospitals.

In this study, we excluded patients who did not meet the following eligibility criteria: available age, gender, surgical approach (open or laparoscopic), and the International Classification of Diseases (ICD) code. Moreover, the LAR must have been performed no more than 3 days after the hospital admission.

### 2.2. Study Variables

The following variables were evaluated for each patient: age, gender, preoperative chemoradiation, ICD code for admission related to the surgery, surgical approach, extent of surgery (multivisceral resection or standard LAR), protective stoma, timing of the procedure (emergency versus elective surgery), in-hospital mortality, hospital LOS, ICU admission, 30-day readmission, antibiotic usage, perioperative blood transfusion, occurrence of AL, and hospitalization costs.

Antibiotic usage was defined as postoperative exposure to a nonprophylactic antibiotic for over 7 days within 30 days of the index surgery, while perioperative transfusion was defined as the use of red cell concentrate within 30 days of the index surgery. AL was considered to be the combination of three conditions: (1) a reoperation or image-guided percutaneous drainage within 30 days of the index surgery; (2) a sign of a clinical AL investigation with at least one postoperative imaging study, such as computed tomography (CT) scan, abdominal X-ray, or other abdominal exams with intravenous contrast; and (3) a sign of a clinical infection investigation with a blood culture exam within 30 days of the index surgery. Only the patients classified as B and C, according to the International Study Group of Rectal Cancer, were included in the AL group [22].

The economic outcomes considered the total hospitalization costs, including 30-day readmissions. These included the cost of materials, pharmacy, transfusion, laboratory and imaging tests, procedures, therapies, inpatient care, and intensive care.

The clinical outcomes were evaluated in terms of both the index surgery admission and any readmission within 30 days. The outcomes that were evaluated and computed in the analysis include in-hospital mortality, hospital LOS, ICU admission, 30-day readmission, antibiotic usage, and AL.

### 2.3. Statistical Analysis

Only cases with no missing values were included in the analysis. Continuous variables were evaluated using Student's *t*-test, and categorical variables were evaluated using the chi-square test or Fisher's exact test. The variables with a *p* value < 0.1 were initially considered for the multivariate analysis. Multivariable Poisson regression analysis was used and presented as a risk ratio (RR) with 95% confidence interval (CI) to determine the factors associated with AL. In order to evaluate costs and LOS, a generalized linear model (GLM) was employed, since it is the preferred approach for multivariate analysis of cost data with gamma distribution [20]. All analyses were two-sided, and a *p* value < 0.05 was considered statistically significant. The statistical analysis was performed using the SPSS (Statistical Package for Social Sciences, version 20.0, Chicago, IL, USA).

## 3. Results

**Figure 1:**
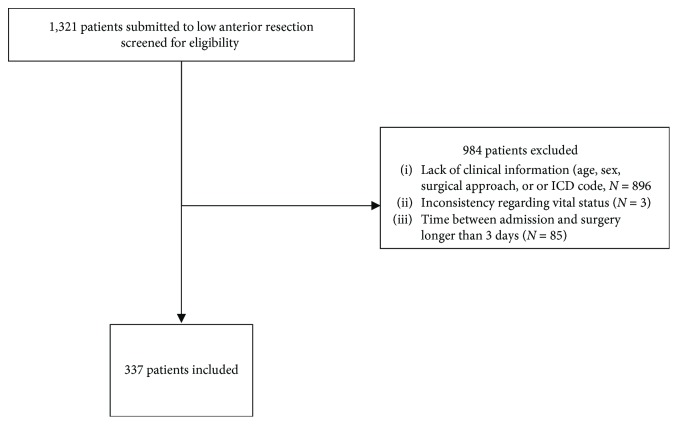
Flowchart of patient selection for the study.

Among the 1,321 potentially eligible patients, 337 fulfilled the eligibility criteria and were included in the analysis (Figure 1). Throughout the index admission, 5.3% of patients fulfilled our criteria for the presence of AL, while considering 30-day readmissions, 1.5% of patients were added, totaling up to 6.8% incidence of AL. Of these, 23 patients developed AL: 1 patient required image-guided percutaneous drainage and 22 required reoperation.

The demographics and perioperative patients' data are presented in Table 1. The mean age was 53.0 ± 16.2 in patients with AL and 51.6 ± 15.4 in patients without AL (*p* = 0.67). The proportion of patients with cancer was higher in patients who developed AL (51.3%) in comparison with those without AL (27.4%) (*p* = 0.016). There was no difference in the use of chemotherapy and radiotherapy among the groups (*p* = 1.00 for both comparisons).

### 3.1. Risk Factors for AL

Table 2 presents an association between possible predictors and AL. Factors such as male sex, cancer diagnosis, emergency surgery, protective stoma, and perioperative blood transfusion increased the incidence of AL in the univariate analysis. No significant differences were found for the procedure extension and surgical approach. When included in the multivariable regression analysis, cancer diagnosis, emergency surgery, and perioperative blood transfusion maintained their significance.

### 3.2. Impact of AL on Clinical Outcomes

Some of the major clinical outcomes are presented in Table 3. Patients with AL showed increased antibiotic usage and higher incidence of readmission and mortality during the follow-up. LOS was also higher in patients with AL (39.6 ± 48.6 days as opposed to 7.5 ± 6.7 days, *p* < 0.001). In the multivariate analysis, including the significant variables (*p* < 0.05), all the analyzed outcomes remained significantly associated with AL. The largest impact of AL was on the mortality rate, which was found to be 13.49 times higher in AL patients than in patients without AL in the analysis adjusted for age, gender, cancer diagnosis, and timing of surgery (*p* < 0.001).

### 3.3. Impact of AL on Economic Outcomes

The economic outcomes of the patients are displayed in Table 4, including data for the whole cohort according to the presence of perioperative AL. The total hospital costs and the index admission costs were found to be greater for patients with AL as compared to patients without AL. The average incremental cost attributable to AL was BRL (Brazilian reais) 170,904 when only index admissions were considered and BRL 175,835 when total hospitalization costs were considered. Readmission costs tended to increase in patients with AL as compared to patients without AL (BRL 6,988 ± 12,965, as opposed to BRL 2,057 ± 10,431, respectively). The increase in costs is attributable to higher service needs. There was not any patient transfer to higher-level medical centers.

In the GLM analysis, the total costs for patients with AL were 6 times higher than those for patients without AL (univariate GLM model coefficient = 6.13; 95% CI: 4.49–8.35, *p* < 0.001). In the construction of a multivariable GLM model that evaluated the total costs, other possible predictors for costs were considered. Multivisceral resection had a *p* value = 0.42. Therefore, it did not enter the multivariable model. The variables selected for GLM were AL, age, gender, cancer diagnosis, timing of surgery, protective stoma, and surgical approach. The coefficients in the multivariable GLM analysis are presented in Table 5. After adjustment for possible confounders, the model showed 4.66 times higher total costs (95% CI, 3.48–6.23, *p* < 0.001) for AL patients than for patients without AL.

## 4. Discussion

AL is a significant cause of postoperative morbidity following colorectal surgery. In this study, we investigated clinical and economic consequences as well as possible predictors for the occurrence of AL with a cohort of patients undergoing LAR for colorectal diseases from a middle-income country, Brazil.

The present study showed that AL, after adjustment for possible confounders, increases the risk of antibiotic usage by approximately 70%, the probability of hospital readmission by three times, and hospital LOS by 4.4 times. Additionally, it has a huge impact on mortality, with a relative risk (RR) of 13.46 (95% CI: 4.10–44.35, *p* < 0.001). These results are consistent with some other reports in the literature, although in our cohort, the strength of association was found to be higher. In a New Zealand cohort of 233 patients undergoing LAR for benign and malignant diseases, patients who developed AL had higher 30-day mortality as compared to patients without AL (6% versus 1%, *p* < 0.05) as well as LOS, which was approximately two times higher in AL patients [19]. In another study conducted in Sweden, which included 6,948 patients who underwent anterior resection, the Odds Ratio (OR) for 90-day mortality was 5.57 (95% CI: 3.29–9.44) when patients with major leaks (when a reintervention was performed) versus those with no leaks were compared [23]. From the economic standpoint, our results show a heavy financial burden associated with AL. The mean costs in the crude analysis was over 6 times higher in the AL group; in the multivariable GLM analysis, which accounted for possible confounders, the association was lower but still significant, with 4.7 times higher total costs (including index admission and 30-day readmission). This can be very challenging in hospitals that are reimbursed by diagnosis-related groups (DRG) or other types of flat-fee agreements, as a high incidence of AL generates substantial expenditure. On the other hand, when the reimbursement is fee for the service, healthcare plans would be financially penalized in case of the occurrence of higher-than-expected AL incidence.

To the best of our knowledge, there are few published papers that evaluate the economic impact of AL. In a North American study with almost 100,000 patients identified through an administrative database, patients with AL encountered hospital expenditures 80% higher than those without leaks in a multivariable analysis adjusted for gender, race, timing (emergency or elective), and type (right or colorectal versus left/sigmoid) of surgery among other variables [20]. The economic impact of AL in the North American study was less than observed in our study, but the North American study included right colectomies that have smaller complication rates and, as a result, lower costs. Another study conducted at English NHS hospitals estimated that the total costs of patients who developed AL after LAR were 2.7 times higher in comparison with those of patients without complications in an unadjusted analysis [6]. However, it is important to keep in mind that the transferability of healthcare-related economic information between countries is not always a straightforward procedure, especially considering local pricing specificities, making it difficult to draw appropriate comparisons between different countries [24].

Our analysis of risk factors for AL did not include a large number of possible predictors, considering the limited amount of information that could be derived from the administrative database. A perioperative blood transfusion, the variable with the highest impact, is an indicator of several factors within the surgical procedure and may indicate anemia, intraoperative significant blood loss, or postoperative adverse events. On the other hand, an emergency procedure, a variable that has not been included in many previous studies on the risk factors for AL, is a strong predictor. Finally, cancer diagnosis (predominantly of the colon and rectum) is also an independent risk factor for AL development.

Some limitations of our study must be acknowledged. The dataset used for all the analyses was based on the patients' administrative information. This approach carries the risk of bias of other retrospective studies as well as the problems associated with the lack of individual clinical patient information, such as the type of anastomosis and tumor distance from the anal verge. A better adjustment in the multivariable analysis could possibly be achieved with more clinical variables, and the definition of AL could be more precise if data from patients' charts was available, for instance. Also, in our multivariable analysis, especially the ones regarding mortality and AL incidence, the number of events was small, and a more robust analysis would demand larger events per covariable proportion. Furthermore, our present study may have underestimated the AL rate. A study conducted with the Swedish Colorectal Cancer Registry showed that the false-negative rate could constitute almost 30% of the AL diagnosis [25]. The occurrence of AL in our study was evaluated only within the 30-day postoperative period, while a study conducted by the Dutch Snapshot Research Group showed that the AL rate could increase by almost 50% beyond 30 days after the surgery [26]. However, considering the incidence of AL grades B and C, which is in line with the other reports in the literature, we believe the risk of AL misclassification to be low. Therefore, the analysis of costs associated with AL is robust, especially the univariate analysis, which does not depend on the deeper verification of possible confounders. Another limitation of our study is the exclusion of over 75% of the original sample secondary to our eligibility criteria, which poses a risk of selection bias. In this regard, it calls attention to the large number of patients with an ICD code of endometriosis. Although this disease does not usually present as an important reason for colorectal surgeries, its rate can be substantial, as seen in a recent French analysis from a national colorectal surgery database [27]. One possible explanation for the high incidence of endometriosis in our sample is that considering that the disease is not frequently associated with colorectal surgeries, the physician might have used this ICD code more accurately than that for other diseases to get approval from the health insurance plans. Since our inclusion criteria required the presence of an ICD code, the incidence of endometriosis in the sample might be secondary to better noting of this information by physicians. Finally, the occurrence of AL and mortality was found to be quite low, and some of the confidence intervals from the estimates were wide (except for the ones from the cost GLM), suggesting that a larger sample would be important to perform a proper adjustment and confirm some of our findings.

In conclusion, AL leads to a heavy burden in LAR procedures from both the clinical and economic perspectives, increasing the hospital costs by 4.66 times. The risk factors identified for the development of AL were emergency surgery, blood transfusion, and cancer diagnosis.

## Figures and Tables

**Table 1:** Demographics and perioperative information of the study patients.

Variable	With AL (*N* = 23)	Without AL (*N* = 314)	*p* value
Male gender	14 (60.9)	123 (39.2)	0.049
Age (years)	53.0 ± 16.2	51.6 ± 15.4	0.670
ICD code			0.093
Gastrointestinal cancers	11 (47.8)	74 (23.6)	
Other cancers^1^	1 (4.3)	12 (3.8)	
Endometriosis	1 (4.3)	44 (14.0)	
Diverticular disease	1 (4.3)	40 (12.7)	
Other^2^	9 (39.1)	144 (45.9)	
Chemotherapy	1 (4.3)	24 (7.6)	1.000
Radiotherapy	1 (4.3)	13 (4.1)	1.000
Emergency surgery	9 (39.1)	48 (15.3)	0.007
Multivisceral resection	9 (39.1)	103 (32.8)	0.647
Laparoscopic surgery	5 (21.7)	118 (37.6)	0.178
Protective stoma	11 (47.8)	45 (14.3)	<0.001
ICU admission	19 (82.6)	158 (50.3)	0.004
Blood transfusion	13 (56.5)	44 (14.0)	<0.001

The sample is comprised of 337 patients. Data is presented as the number of patients (%) except for age, which is expressed as mean ± standard deviation. ICU: intensive care unit; ICD: International Classification of Diseases. ^1^There were 11 cases of cancer in other localizations (3 cervical cancers, 5 ovarian cancers, 2 prostate cancers, and 1 uterine cancer) and 2 unspecified cancer codes. ^2^An unspecific diagnosis such as the acute abdomen, bowel abscess, inflammatory bowel disease, bowel motility disorders, abdominal pain, hemorrhage, intra-abdominal mass, benign tumors, intestinal obstruction, peritonitis, and bowel volvulus.

**Table 2:** Univariate and multivariable analyses of the possible predictors of AL.

Variables	AL incidence	Crude RR (95% CI)	*p*	Adjusted RR (95% CI)	*p*
Gender					
Male	14 (10.2)	2.27 (1.01–5.09)	0.049	1.72 (0.82–3.62)	0.148
Female	9 (4.5)				
Surgery timing					
Emergency	9 (15.8)	3.16 (1.44–6.94)	0.007	2.56 (1.15–5.71)	0.021
Elective	14 (5.0)				
Procedure extension					
Multivisceral resection	9 (8.0)	1.29 (0.58–2.89)	0.647	—	—
Standard LAR	14 (6.2)				
Surgical approach					
Open	18 (8.4)	2.07 (0.79–5.43)	0.178	—	—
Laparoscopic	5 (4.1)				
Blood transfusion					
Yes	13 (22.8)	6.38 (2.94–13.84)	<0.001	4.44 (1.85–10.63)	0.001
No	10 (3.6)				
Protective stoma					
Yes	11 (19.6)	4.59 (2.13–9.89)	<0.001	1.88 (0.74–4.80)	0.185
No	12 (4.3)				
Cancer diagnosis					
Yes	12 (12.2)	2.66 (1.22–5.83)	0.017	2.51 (1.27–4.98)	0.008
No	11 (4.6)				

Incidence is presented as *N* (%). *p* value was calculated by Fisher's exact test in the crude analysis with Poisson regression in the multivariable analysis. The multivariable Poisson regression included variables with *p* < 0.10 in univariate analysis: gender, surgery timing, cancer, and need for blood transfusion. AL: anastomotic leak; CI: confidence interval; RR: relative risk.

**Table 3:** Clinical outcomes according to the presence of AL.

Outcomes	With AL (*N* = 23)	Without AL (*N* = 314)	Crude RR (95% CI)	*p*	Adjusted RR (95% CI)	*p*
Use of antibiotics	20 (87.0)	146 (46.5)	1.87 (1.54–2.28)	<0.001	1.69 (1.37–2.09)	<0.001
Mortality	5 (21.7)	4 (1.3)	17.06 (4.91–59.25)	<0.001	13.49 (4.10–44.35)	<0.001
Readmission	6 (26.1)	26 (8.3)	3.15 (1.44–6.87)	0.014	3.34 (1.53–7.32)	0.003
LOS	39.6 ± 48.6	7.5 ± 6.7	5.20 (3.91–6.90)	<0.001	4.42 (3.38–5.78)	<0.001

Data is presented as *N* (%), except for LOS, which is presented as mean ± standard deviation. Crude RR was calculated from contingency tables, except for the LOS, where a generalized linear model (GLM) was used (both in the crude and in the adjusted RR). Adjusted RR (except for LOS) was calculated using Poisson regression. Adjusted analyses included age, gender, cancer status, and timing of surgery as covariables (except for LOS, where gender was omitted in order to achieve model convergence). *p* values were obtained by Fisher's exact test and Poisson regression for all variables except for LOS, which was calculated with GLM. AL: anastomotic leak; CI: confidence interval; LOS: length of stay; RR: relative risk.

**Table 4:** Cost outcomes (Brazilian R$ or BRL) for the whole cohort according to the presence of AL.

Time period	Whole cohort	With AL (*N* = 23)	Without AL (*N* = 314)	*p* value
Index admission	43,878 ± 83,265	203,117 ± 242,601	32,213 ± 36,136	0.003
Readmissions	2,393 ± 10,673	6,988 ± 12,965	2,057 ± 10,431	0.087
Total costs	46,271 ± 83,675	210,105 ± 238,091	34,270 ± 37,613	0.002

Values are expressed as mean ± standard deviation. AL: anastomotic leak; IQR: interquartile range. *p* value was calculated using Student's *t*-test.

**Table 5:** GLM for total costs (index admission plus readmissions).

Variable	Crude estimate	95% CI	*p*	Multivariable estimate	95% CI	*p*
Anastomotic leak	6.13	4.49–8.35	<0.001	4.66	3.48–6.23	<0.001
Emergency surgery	1.81	1.40–2.32	<0.001	1.25	1.01–1.54	0.038
Age	1.02	1.01–1.02	<0.001	1.01	1.01–1.02	<0.001
Male gender	1.47	1.21–1.78	<0.001	0.87	0.74–1.01	0.070
Laparoscopy	0.683	0.56–0.83	<0.001	1.29	1.11–1.52	0.001
Cancer	1.54	1.25–1.90	<0.001	1.29	1.09–1.52	0.002
Protective stoma	3.15	2.50–3.97	<0.001	1.91	1.54–2.36	<0.001
Intercept	—	—	—	14,221	10,739–18,832	<0.001

Multivariable estimates were adjusted for all the variables presented in this table.

## Data Availability

The dataset is available from the ResearchGate repository (DOI: 10.13140/RG.2.2.13952.69128). Further information is available from the corresponding author on request.
